# Supplementary material for: Iterative improvement in the automatic modular design of robot swarms
Source: PeerJ Comput Sci. 2020 Dec 7;6:e322. doi: 10.7717/peerj-cs.322 (PMC7924708; doi:10.7717/peerj-cs.322)
Supplement: Supplemental Information 3 [file peerj-cs-06-322-s003.zip › argos3/doc/api/standalone/a00630_source.html]

ARGoS: plugins/simulator/visualizations/qt-opengl/models/qtopengl\_box.h Source File


- Main Page
- Related Pages
- Namespaces
- Classes
- Files

- File List
- File Members

# plugins/simulator/visualizations/qt-opengl/models/qtopengl\_box.h

Go to the documentation of this file.

```
00001 
00007 #ifndef QTOPENGL_BOX_H
00008 #define QTOPENGL_BOX_H
00009 
00010 namespace argos {
00011    class CQTOpenGLBox;
00012    class CBoxEntity;
00013 }
00014 
00015 #ifdef __APPLE__
00016 #include <gl.h>
00017 #else
00018 #include <GL/gl.h>
00019 #endif
00020 
00021 namespace argos {
00022 
00023    class CQTOpenGLBox {
00024 
00025    public:
00026 
00027       CQTOpenGLBox();
00028 
00029       virtual ~CQTOpenGLBox();
00030 
00031       virtual void DrawLEDs(CBoxEntity& c_entity);
00032       virtual void Draw(const CBoxEntity& c_entity);
00033 
00034    private:
00035 
00036       void MakeBody();
00037       void MakeLED();
00038 
00039    private:
00040 
00041       GLuint m_unBaseList;
00042       GLuint m_unBodyList;
00043       GLuint m_unLEDList;
00044       GLuint m_unVertices;
00045 
00046    };
00047 
00048 }
00049 
00050 #endif
```

---

Generated on 10 Jul 2018 for ARGoS by 
 1.6.1
